# Optical Biosensors—Principles of Operation and Applications

**DOI:** 10.3390/mi17050579

**Published:** 2026-05-07

**Authors:** Tomasz Blachowicz, Guido Ehrmann, Elzbieta Stepula, Andrea Ehrmann

**Affiliations:** 1 Institute of Physics—Center for Science and Education, Silesian University of Technology, 44-100 Gliwice, Poland; tomasz.blachowicz@polsl.pl; 2 Virtual Institute of Applied Research on Advanced Materials (VIARAM);; 3 Faculty of Engineering and Mathematics, Bielefeld University of Applied Sciences and Arts, 33619 Bielefeld, Germany; elzbieta.stepula@hsbi.de

**Keywords:** portable biosensors, point-of-care diagnosis, optical sensors, microfluidics

## Abstract

Biosensors have a recognition element that detects a bioanalyte as well as a transducer that transfers the measured physicochemical properties into an electric signal, which is amplified, processed, and depicted on a user interface and usually stored in a data storage system. Such biosensors can be used in a broad range of applications, from personalized medicine to drug discovery, and from food safety to plant disease diagnosis. Portable biosensors are often based on microfluidic systems or micro-electromechanical systems (MEMS), measuring physical or chemical parameters. In spite of their importance for diverse applications, there are still several limits regarding the portability of biosensors, which is often necessary. Besides the required miniaturization of the components and the limited lifetime of some biological reagents, sample preparation and handling can be problematic. This review gives an overview of recent biosensor research, concentrating on optical measurements, and shows the possibilities and limits of the biosensors developed during the last few years.

## 1. Introduction

Portable biosensors have a wide range of applications. In medicine, they can be used for long-term monitoring of elderly and chronically ill people [1,2,3] as well as for athletes [4,5] or firefighters on duty [6,7], but also in point-of-care diagnosis [8,9,10]. Inexpensive and easy-to-use biosensors are especially relevant for global healthcare [11,12]. Besides medical applications, there are many more fields in which biosensors are used, such as drug discovery [13,14,15], forensics [16,17,18], and detection of biological warfare agents [19,20]. Biosensors can also be used for food safety [21,22,23], in the fermentation industry [24,25], or for the diagnosis of plant diseases [26,27,28].

There are many different types of biosensors, e.g., those based on microfluidics [29,30] or micro-electromechanical systems (MEMS) [31,32] and those working with various physical or chemical principles [33,34,35]. A large area of research and implementation is based on optical biosensors, applying colorimetry [36], fluorescence [37], chemiluminescence [38], spectroscopy [39] or using a microscope [40]. This review gives an overview of such optical biosensors for diverse applications and explains their potential as well as recent limits and future challenges, focusing on original research papers from recent years and pointing out suitable review papers regarding older research and adjacent topics.

It should be mentioned that biosensors generally contain a biorecognition element, a transducer, and a signal processing unit, which differentiates biosensors from standalone analytical techniques, such as Raman spectroscopy, Fourier-transform infrared spectroscopy, or UV/Vis spectroscopy. As opposed to general chemical characterization, the analytical techniques serve as the readout methods for the detection of the specific analyte recognized by the recognition unit.

This review provides a unified performance and translation-oriented framework, connecting physical principles of optical biosensors with different types of transducers, and performance metrics with real-world problems impeding the commercialization of many techniques. Thus, it does not focus only on a single technique, application, or material class. Recently, no review has been available comparing several performance metrics of all major optical transducers. In addition, this review describes the whole chain, from physical principles to devices to possible applications, including various domains of applications and not only recent techniques, but also classical ones, to give the broadest possible overview.

Here, we present and review the results of our literature search performed in February/March 2026, concentrating on papers indexed in the Web of Science, supplemented by papers found in Google Scholar, mainly from the years 2022–2026. Besides general keywords such as “optical biosensors”, several specific transducers and applications were researched, all of which are given in this review as subsection headers. The combination of a general search with more specific keywords leads to a comprehensive overview given in this review.

Therefore, a mechanism-to-application translational framework for optical biosensors is given here, providing the full perspective from sensing principles to real-world use.

## 2. Biosensors

**Figure 1:**
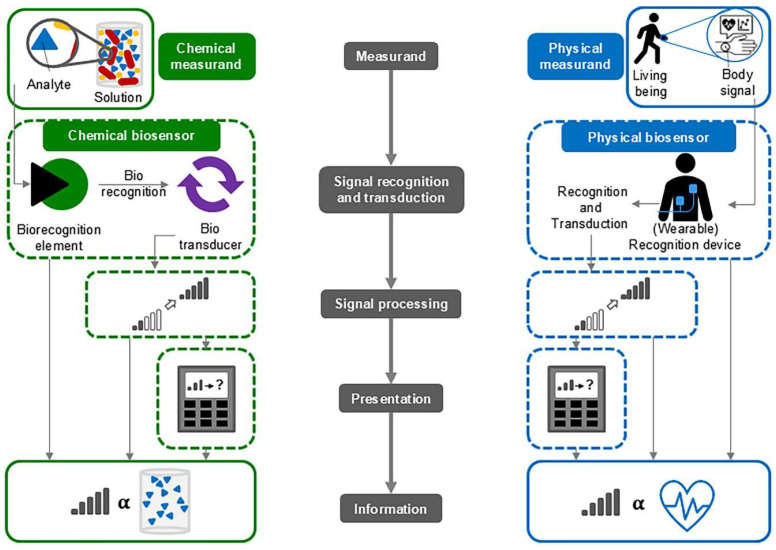
Schematic illustration of chemical (**left**) and physical (**right**) biosensors—structure and working principles. From [44], originally published under a CC-BY license.

The definition of biosensors varies slightly in the literature, but usually biosensors consist of a bioreceptor, a transducer that converts the biological response into a signal, a signal-processing system that detects the signal, and a user interface [41,42,43]. While most authors define biosensors as chemical sensors, there are also definitions of biosensors measuring physical signals [44], such as wearable sensors for health monitoring [45,46,47,48], implantable sensors for blind people [49,50,51], or other sensors detecting biosignals. These two measuring paths—chemical and physical biosensors—are depicted in Figure 1 [44].

### 2.1. Physical Biosensors

Physical biosensors are scarcely mentioned in the literature. Typical physical parameters that can be measured include ECG and pulse, breathing rate, blood pressure, intraocular pressure, body temperature, the number of steps taken per day, and eye movements [44]. Some authors generalize the term “biosensor” without differentiating between chemical and physical biosensors [52] or concentrate on the description of the measured parameter [53,54,55]. While this review mainly concentrates on chemical biosensors, a few physical biosensors based on optical principles are also mentioned, especially photoplethysmography.

### 2.2. Chemical Biosensors

Chemical biosensors incorporate a recognition element that selectively binds the target analyte. This recognition element may be a biorecognition element (e.g., enzyme, antibody, microorganism, cell, or nucleic acid) or a synthetic/biomimetic recognition element (e.g., molecularly imprinted polymer or aptamer) [42,44]. The sensor can be implemented as a wearable device [56,57,58], can use microneedles [59,60,61], can be implanted [62,63,64] or can be used at the point-of-care [65,66,67], with further possibilities for non-medical applications.

Enzymes have strong catalytic functions and relatively high substrate specificity, which makes them suitable for sensing or detecting target analytes in biological systems [68]. In enzyme-based electrochemical biosensors, e.g., enzymes are immobilized on electrodes, which can be achieved by nanomaterial modification, applying metal or metal-oxide nanoparticles, graphene or carbon nanotubes, metal–organic frameworks, or conductive polymers [69]. Such enzymatic biosensors can be used to detect small chemical molecules, especially by using metalloenzymes with metal ions in their core structure [70].

Natural or synthetic antibodies can be used, e.g., to detect cancer cells [71], kidney injury [72], food toxins [73] or gluten [74]. Generally, such antibody-modified electrodes can be used as biosensors due to their affinity for antigens, which are used, among other applications, in the well-known enzyme-linked immunosorbent assay (ELISA), where primary antibodies are immobilized on a solid surface and capture a specific antigen in the sample proteins [75].

Microbial biosensors can be used for measuring biochemical oxygen demand (BOD), which is a main indicator for water quality [76,77,78]. For this purpose, single-chamber biosensors are often used that contain a bio-anode inside the main reactor compartment, where electroactive bacteria catalyze the oxidation of organic pollutants in wastewater, and an air-diffusion cathode on the side of the reactor in air, where oxygen from the air works as an electron acceptor. The potential difference between both electrodes enables measuring the current over an external load as a measure of the BOD concentration [77].

Cell-based biosensors, with living cells as sensing elements, can be used, e.g., for food safety [79], environmental monitoring [80], and bisphenol A detection [81]. Generally, whole-cell biosensors (WCBs) have a high resistance to environmental changes due to the metabolic regulation ability of living cells and are relatively easy and inexpensive to prepare, making them advantageous for suitable applications [79].

DNA-based biosensors can be subdivided into functional DNA-strand-based, DNA hybridization-based, and DNA-templated biosensors and also have relatively low production costs, combined with long lifetimes and wide detection targets [82]. They are often used for the clinical diagnosis of disease biomarkers [83], cancer or cardiovascular health [84]. Similarly, RNA-based biosensors are also often used in clinical applications [85,86].

Besides these biorecognition elements, many more can be used to define the specificity of a reaction with the target analyte.

### 2.3. Transducers

The transducer is responsible for the conversion of a biochemical reaction between the target analyte and the bioreceptor—or a physical effect in the case of physical biosensors—into a measurable readout [44].

Electronic transducers are often used, e.g., for measuring blood glucose levels. Among the electrochemical biosensors, there are amperometric, voltammetric, impedimetric, capacitive, potentiometric, photoelectrochemical or electrochemiluminescent sensors. Such sensors with different kinds of electronic transducers can be used for health monitoring and point-of-care diagnostics, as they are portable and can be integrated into wearables or even implanted [87,88,89].

Thermal or calorimetric transducers measure the thermal energy emitted or absorbed due to a biological response [90]. Usually, the conversion of a substance by a catalyst is accompanied by a heat release, as most biochemical reactions are exothermic [91]. By integrating single thermocouples into microchannels, small-volume microfluidic biosensors can be produced, enabling new paths for lab-on-a-chip diagnostics [92].

Acoustic or piezoelectric transducers are based on the transmission and reception of acoustic pressure waves [93]. Among the acoustic biosensors, there are ultrasound, electroacoustic, magnetoacoustic, thermoacoustic, photoacoustic, and other sensors [93]. While acoustic biosensors can be physical sensors, directly measuring parameters of the human body, e.g., by ultrasound [94], acoustic transducers can also be used for immunosensing [95] or in combination with electrochemical sensors, where surface acoustic waves improve the sensor sensitivity and response rate [96].

Finally, optical transducers, which are the focus of this review and include those based on surface plasmon resonance (SPR), adsorption or reflection, luminescence, fluorescence, optical fibers and others, will be described in detail in the next section.

## 3. Biosensors with Optical Transducers

Optical transducers are based on the optical interaction between the recognition element and the bioanalyte [97]. Optical transducers can be based on different principles, e.g., colorimetry, fluorescence, and intensity of reflected light in different wavelength ranges, spectrometry. In photoplethysmography, a direct optical measurement on the skin is used to measure vital signs [98], i.e., this technique belongs to the physical biosensors, while the other biosensors mentioned here are chemical biosensors. In some cases, optical fibers, lens systems or other optical elements are integrated to improve the detection of the optical signal. This section gives an overview of the most recent developments in optical transducers.

Due to the broad range of optical transducers and the inclusion of photoplethysmography as a physical biosensor, this section is sequentially structured to avoid jumping between different techniques and an artificial classification system, especially since some techniques cannot be clearly classified, e.g., UV/Vis spectroscopy being used for labeled and label-free detection.

### 3.1. Photoplethysmography

**Figure 2:**
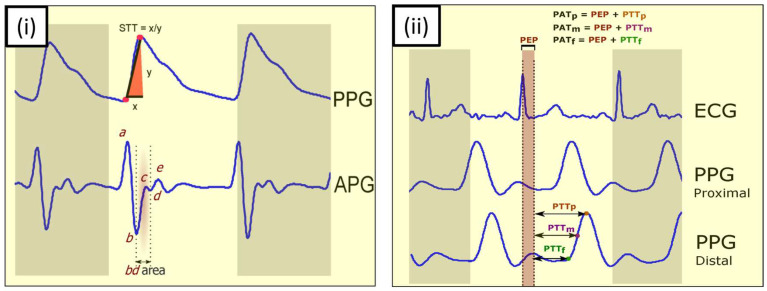
Key features of blood pressure estimation using PPG and other physiological signals. (**i**) Using the PPG signal and its derivative; (**ii**) using ECG and PPG signals. PPG—photoplethysmogram, APG—acceleration photoplethysmogram, STT—slope transit time, PTT—pulse transit time. From [102], originally published under a CC-BY license.

Photoplethysmography (PPG) is a well-established technique dating back approximately one century. Pulse oximetry measures light absorption by tissue (typically at the fingertip) over time to estimate heart rate and arterial oxygen saturation (SpO_2_). A PPG sensor consists of a light source, usually an LED, and a photodetector. The light emitted from the LED is reflected from the tissue of the human body. The PPG measurement is based on the principle that blood flowing through arteries absorbs more light, i.e., reduces the reflected light measured by the photodetector [99]. The pulsatile waveform thus allows measuring the heart rate, as already depicted in Figure 2. From the time-dependent heart rate, the heart rate variability and sleep quality can be estimated, while blood pressure and blood oxygen saturation need more sophisticated algorithms, as mentioned above [100].

In addition, the plethysmographic signal may show respiration- or ventilator-induced modulations, especially in mechanically ventilated patients [101]. PPG detects the change in blood volume in transmission or reflection mode [102]. The pulsatile component of the PPG is synchronous with the heartbeat, while the non-pulsatile component correlates with the baseline blood volume, respiration, sympathetic nervous system, and thermoregulation [103]. Figure 2 shows some key features of blood pressure estimation and correlations of the acceleration photoplethysmogram (APG) with the ECG [102].

The evaluation of blood pressure based on the PPG is not easy. However, correlations with the slope transit time (STT in Figure 2i), i.e., the steep trend of the rising pulse wave [104], or the b-d area marked red in Figure 2i [105] have been shown. In combination with the ECG, the pulse arrival time (PAT; Figure 2ii) and pulse transition time (PTT in Figure 2ii) can be measured from the PPG.

As already mentioned, PPG is a quite old and well-established technique. Nevertheless, there are regular reports of advances in integration in wearables, novel sensors, and new applications. The integration of PPG measurements into wearables is mostly done by using wrist-wearable devices, where PPG sensors can measure the pulsatile blood flow in the arteries of the wrist [99].

Similar to ECG measurements by textile electrodes [48], movements of the user can also influence PPG measurements by wrist sensors, making them potentially inaccurate and not fully reliable [106]. Other challenges of wearable PPG sensors are related to energy consumption, a common problem in wearables, and miniaturization [104].

**Figure 3:**
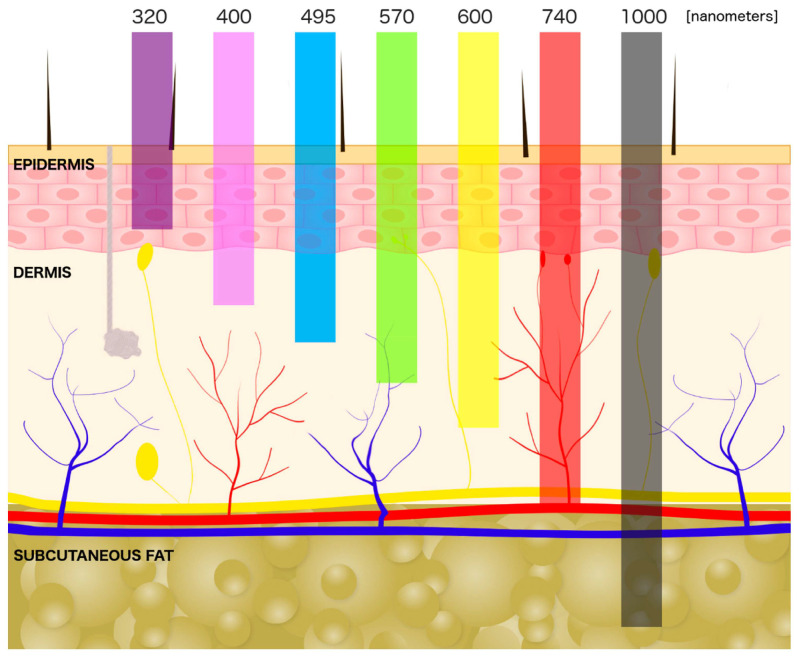
The skin cross-section showing dermal penetration by different wavelengths of light (in order from the left: UVB, UVA, blue light, green light, yellow light, red light, infrared light). From [107], originally published under a CC-BY license.

An important parameter in PPG measurements is the wavelength of the LED used. Generally, the light penetration depth into the skin varies with the wavelength, as depicted in Figure 3 [107]. Shorter wavelengths (blue–green) are absorbed and scattered more strongly and probe mainly superficial layers, whereas longer wavelengths (red to near-infrared) penetrate more deeply into the dermis and subcutaneous tissue. This is why red and infrared light are often used in medical equipment, as they measure the peripheral oxygen saturation (SpO_2_) most accurately, while green light is often used in wearables with PPG sensors, as such sensors are less affected by motion artifacts [108]. With two different green wavelengths, not only the pulse rate, but also oxyhemoglobin saturation can be measured [108]. Green light, on the other hand, has a limited skin penetration on dark-skinned people, necessitating a higher light intensity to improve the accuracy [109]. Other factors, like obesity, can also strongly influence the measured signal [110], suggesting further research regarding the impact of diverse factors on the PPG signal. It must be mentioned that many more factors are known to influence the PPG signal, such as age, sex, body temperature, sweat, hair and tattoos [109,111].

Besides these factors, the accuracy of PPG data naturally depends on the quality of the sensor and the sensor position, often leading to less accurate results measured by commercial wrist-worn fitness trackers [112]. The most straightforward data, heart rate and heart rate variability, are usually measured accurately [113], while arterial stiffness or blood pressure are usually measured with clinical PPG equipment [114,115,116].

Approaches to further improve the accuracy of PPG measurements include the use of deep learning methods to improve PPG signal analysis [117] or ultra-low-power PPG [118]. Finally, it should be mentioned that besides the common PPG method, nowadays many researchers are working on remote photoplethysmography (rPPG). This technique uses cameras to detect physiological changes based on measuring small skin color variations upon blood flow, as described in detail in Ref. [119].

### 3.2. Colorimetry

#### 3.2.1. Nanoparticle-Based Colorimetry

Colorimetry, not to be confused with the aforementioned calorimetry, is used to detect reactions with analytes by color changes [120,121]. These color changes can be induced by different principles.

**Figure 4:**
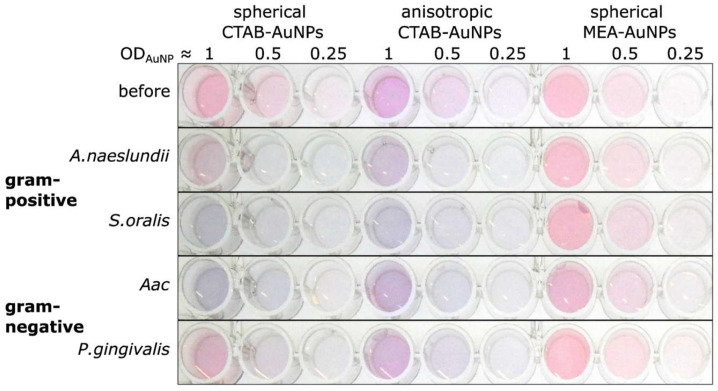
A representative image of a well plate containing different concentrations of spherical and anisotropic gold nanoparticles (AuNPs) with different surfactants, Cetyltrimethylammoniumbromid (CTAB) and mercaptoethylamine (MEA), before and 10 min after the addition of oral bacteria (*A. naeslundii*, *S. oralis*, *Aac* and *P. gingivalis*). From [125], originally published under a CC-BY license.

Among them, the localized surface plasmon resonance (LSPR) has recently gained interest. Localized surface plasmons are the coherent oscillations of free electrons relative to the positively charged lattice in metal nanoparticles, excited by electromagnetic radiation. This resonance produces enhanced absorption and scattering (extinction) at a wavelength that depends on particle size, shape, material, and the surrounding dielectric environment [122,123]. Typical materials for such nanoparticles are gold and silver, both with their own advantages and disadvantages [124]. An example of an LSPR-based measurement of four different oral bacterial species with differently functionalized gold nanoparticles of different shapes is given in Figure 4 [125].

A similar measurement method is based on coupled LSPR (c-LSPR), whose resonance conditions are red- or blue-shifted (i.e., have lower or higher energy) in comparison to the corresponding single-particle LSPR wavelengths and occur due to the formation of nanoparticle dimers, which can have bonding and antibonding hybrid plasmon modes [126]. This effect can be created by equivalent or non-equivalent nanoparticles, which react differently to the incident light [127]. While this technique in general enables producing colorimetric biosensors that can be finetuned in any required way, the usual techniques to prepare the necessary nanostructures are relatively complex, expensive and time-consuming, making this approach only useful for specific applications where other methods are insufficient [128,129]. This is why several authors investigated low-cost approaches, such as using a polystyrene-bead template to prepare a hexagonal lattice [130] or self-assembled colloidal nanoparticle arrays as template masks [131].

Another colorimetric method is based on surface lattice resonance (SLR), where metallic nanoparticles on a periodic lattice diffract light if the incident radiation wavelength is similar to the lattice constant, and these diffractive waves couple with single-particle localized surface plasmons, resulting in so-called lattice plasmon modes [132]. The SLR generally has lower energy than the corresponding LSPR [132]. The reaction to the incident light depends on the irradiation angle with respect to the surface; the best way of illumination is the attenuated total reflection (ATR) geometry, where an array of nanoparticles is illuminated by total reflection in the transparent substrate [133]. For this method, a high lattice periodicity is necessary to obtain SLR of high quality [134].

**Figure 5:**
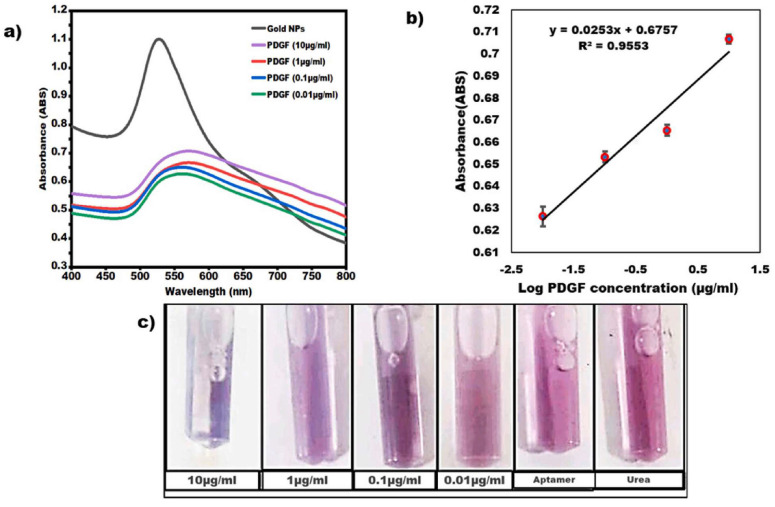
Different concentrations of Au NPs based on the colorimetric method. (**a**) UV-spectrophotometer shows the absorption of Au NPs alone and platelet-derived growth-factor (PDGF)/AptPDGF/Au NPs (10 μg/mL, 1 μg/mL, 0.1 μg/mL and 0.01 μg/mL). Aptamer introduced in the presence of PDGF. (**b**) Error bar represents the standard deviation of the assay for each concentration, repeated 5 times. (**c**) Naked eye observation of PDGF at various concentrations introduced with aptamer and GNPs. Various concentrations of platelet-derived growth factors were added to plastic vials (2 mL) containing 26 μL of aptamer (10 μM). After incubation for 10 min, 596 μL of the gold nanoparticles was added. After reacting for 5 min, 317 μL of NaCl (0.25 M) solution was transferred rapidly into the vials. After incubation for the next 5 min, the resulting solution was transferred for UV-VIS spectroscopy. From [136], originally published under a CC-BY-NC-ND license.

Besides these platform-based colorimetric techniques, there are also techniques based on colloidal solutions of nanoparticles, based on the same physical principles, which can usually be prepared more easily and cost-effectively, while their results are less exact [135]. The optical response of colloidal nanoparticle solutions depends mostly on the LSPR modes of the single nanoparticles, i.e., on their size and shape distribution [135]. Often, colloidal nanoparticles are labeled (functionalized) with ligands that can bind to the analytes, resulting in aggregation of the labeled nanoparticles via the analytes or anchoring of the nanoparticles on large analytes, both leading to the aforementioned LSPR shift [135]. As an example, Figure 5 depicts the absorbance of gold nanoparticles alone and with different concentrations of platelet-derived growth factor (PDGF) [136].

Label-free colorimetric biosensors, i.e., biosensors that use the analyte’s inherent material qualities such as size, surface charge, and refractive index, use electrostatic forces or hydrogen-bonding to support aggregation of the nanoparticles due to the analytes [137]. An often-used method is the adsorption of negatively charged aptamers, resulting in dispersed negatively charged aptamer-nanoparticles, from which the aptamer can be detached by the analyte [138].

While the aforementioned colorimetric sensors are based on color changes upon agglomeration of nanoparticles, it is also possible to create chemical reactions that lead to color changes, e.g., by artificial nano-enzymes (so-called nanozymes) that may be used to oxidize a substrate [139], by target-mediated oxidation of noble metals such as Ag [140], or by the growth of metal seeds mediated by enzymes or reducing agents [141].

#### 3.2.2. Enzyme-Based Colorimetry

While most colorimetric techniques nowadays are based on nanoparticles, other techniques have also been reported in the literature.

**Figure 6:**
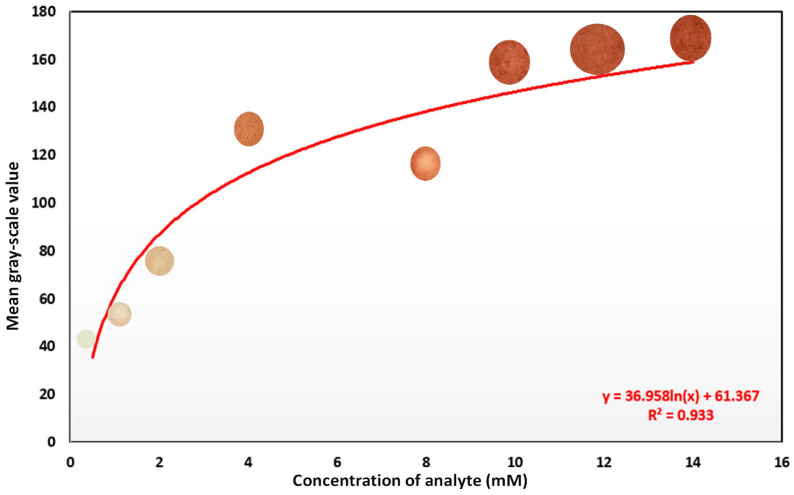
Response curve of the spot-based glucose assay. From [145], originally published under a CC-BY license.

Among them, enzyme-based colorimetry, enabled by enzymes’ catalytic properties, shows high sensitivity and selectivity [142,143,144]. As an example, Figure 6 shows an enzyme-based glucose assay that could be evaluated by a smartphone [145].

On the other hand, enzymes are problematic to use in varying environmental conditions as they are modified by temperature and other parameters [146]. This led to the approach of using metal–organic frameworks (MOFs) to immobilize enzymes, in this way combining the enzymatic activity with stabilized behavior under varying environmental conditions [147,148]. Enzymes can also be immobilized in chitosan films by glutaraldehyde, making the enzyme more stable and highly selective [149].

While some other colorimetric biosensors can be found in the literature, often mimicking natural enzymes [150,151,152], nanoparticle-based, nanozyme-based and enzyme-based approaches are most often reported nowadays.

### 3.3. Spectrophotometry

Different spectrophotometric methods can be used for spectrophotometric biosensors, including measurements with ultraviolet/visible (UV/Vis) spectrophotometers, measurements in the near-infrared (NIR), and Raman spectrometry.

UV/Vis spectrophotometry is usually used as the readout method for the aforementioned colorimetric sensors with nanoparticles or enzymes [153,154]. However, this technique is mostly used for validation of results that are also visible with the human eye [136,155].

On the other hand, UV/Vis spectrophotometry can also be used directly to measure the quality characteristics of fruit juice, as several researchers have shown [156,157]. Measurements of fruit can also be performed in the NIR [158,159] or in a combination of visible light and NIR [160,161,162].

Different spectroscopic techniques can be used to detect viruses. Raman spectroscopy uses inelastic scattering to detect polar and non-polar bonds in a sample without the necessity of a reagent [163]; nevertheless, many authors define this technique as an optical biosensor. It can be used to detect a broad range of viruses [164,165,166]. NIR and Fourier-transform infrared (FTIR) spectroscopy can also detect biomolecules such as proteins, lipids, and carbohydrates [167], enabling the detection of viruses [168,169,170].

Raman spectroscopy is a powerful technique often used for label-free detection of analytes, as it provides unique vibrational fingerprints of molecules. However, conventional Raman scattering is inherently weak, making it challenging to detect very low analyte concentrations. To overcome this limitation, surface-enhanced Raman spectroscopy (SERS) is widely employed in biosensing. In SERS-based sensors, metallic nanostructures or nanoparticles—typically gold or silver—are functionalized with Raman-active reporter molecules and biorecognition units (such as antibodies, aptamers, or peptides). These components create intense electromagnetic “hot spots” that greatly amplify Raman signals, enabling sensitive and selective detection even at trace-level analyte concentrations [171]. SERS-based biosensing has rapidly expanded in scope, enabling ultrahigh-sensitivity detection of diverse biological targets. For example, SARS-CoV-2 viral particles have been detected directly from clinical samples using plasmonic substrates and SERS nanotags, while protein and nucleic acid biomarkers can be quantified at femtomolar levels in complex matrices [172]. Deng et al., e.g., used polarization-differential spectrophotometry with a high-performance single-mode green fiber laser to increase the sensitivity for the detection of trace hemoglobin by two orders of magnitude, as compared with conventional spectrophotometry [173]. Baptista et al. concentrated on 16 specific optical wavelengths, combined with different post-processing algorithms, to detect even low-level infections with *Plasmodium falciparum* for accurate malaria diagnosis [174]. Hilner et al. suggested a relatively simple method of pH adjustment by phenol red spectrophotometry in a plate reader to increase the number of protocols in tissue engineering and other experiments where the pH value, as an important parameter for cell growth, is mentioned [175]. Guo et al. combined UV-spectrophotometry with electrochemical detection to detect the lethal mycotoxin zearalenone (ZEN) with improved accuracy [176].

**Figure 7:**
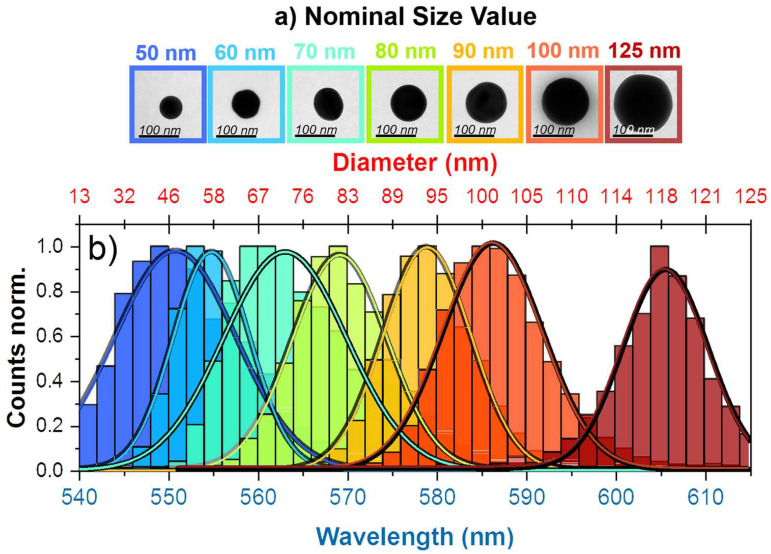
(**a**) TEM images of monomers with different nominal sizes. (**b**) Histogram distributions of the plasmon resonance peak of all seven nanoparticle lots are characterized; the histogram’s top axis has been converted to nanoparticle diameter. Each histogram has been fitted with a Gaussian distribution (solid line). From [177], originally published under a CC-BY license.

Finally, a slightly different spectrophotometric method should be mentioned that does not directly detect biological targets but measures the diameter distribution of gold or other nanoparticles that can be used for colorimetric biosensors. For this, Calvo et al. developed a method called dark-field single particle spectrophotometry that is based on the spectral analysis of the nanoparticle light scattering by using a microscope with white light from a halogen lamp and an electro-optical filter, enabling tuning the wavelengths of the light that is focused on the sample surface through a beam-splitter and a dark-field objective [175]. In this way, the diameters of round nanoparticles could be measured unambiguously with high accuracy, as depicted in Figure 7 [177].

### 3.4. Polarimetry

While most of the aforementioned optical methods use unpolarized light, there are also techniques based on the application of polarized light. Sharma et al. used polarization-modulated spectroscopic ellipsometry and wide-field Mueller matrix polarimetry to detect *E. coli* bacteria in microfluidic applications [178]. A polarimetric waveguide interferometer was presented by Hörmann et al., who used edge couplers to excite different polarizations from an optical fiber, combined with a linear polarizer to measure the phase information, and integrated the system into a biosensing platform [179].

**Figure 8:**
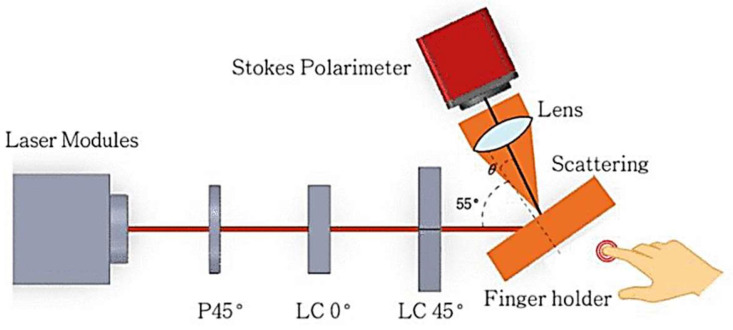
Schematic illustration of Mueller matrix polarimetry setup, with a linear polarizer P45° and two liquid-crystal variable retarders LC0° and LC45°. From [183], originally published under a CC-BY license.

A polarimetric biosensor for non-invasive glucose monitoring was suggested by Kopp, who measured the glucose-induced optical rotation by a GeSe photodetector on a polyethylene terephthalate (PET) substrate [180]. Generally, polarimetry is often used for glucose monitoring, as the chiral glucose molecules lead to different complex refractive indices when exposed to left- and right-handed circularly polarized light [181]. Thus, the rotation of linearly polarized light is proportional to the concentration of glucose (or other chiral) molecules [181]. To improve the sensitivity, polarization modulation is often used with magneto-optic modulators, liquid-crystal modulators, a chopper, etc. [181,182,183]. Figure 8 shows an example of a non-invasive glucose test with a polarimeter and a polarizer (P) as well as two liquid-crystal (LC) variable retarders to prepare linear polarization states at 0°, 45°, and 90°, as well as a right-hand circular polarization state [183].

### 3.5. Luminescence Detection

Using luminescence for biosensing and bioimaging has the advantage that autofluorescence signals from tissues and potential long near-infrared afterglow emissions are removed [184]. For this purpose, persistent luminescence nanoparticles such as gold, quantum dots, magnetic nanoparticles, or carbon nanostructures, combined with suitable biosensors such as aptamers, antibodies, or enzymes, can be used [184].

In chemiluminescence-based biosensors, the luminescence light stems from a biochemical reaction between the target analyte and the biorecognition element, when the excited molecule returns to its ground state without the necessity for an external light source [185]. Chemiluminescence can be detected by a photomultiplier tube over a wide linear dynamic range and can be effectively used in miniaturized analytical systems, e.g., microarrays or microfluidics [186]. For example, for the detection of multiple toxins, an assay with an optical fiber aptasensor system, i.e., with a single-stranded binding protein and mycotoxin aptamers for selective analysis, was suggested, where the optical fibers carried the immobilized single-stranded binding protein and also transduced the chemiluminescent emission [187]. Similar optical fiber-based chemiluminescent biosensing platforms have been reported by several research groups [188,189,190].

Bioluminescence arises from biological systems, e.g., proteins and organisms, and leads to higher quantum yields than common chemiluminescence [191]. Most often, it is based on the luciferin-luciferase enzymatic reaction in living cells [192]. It is also possible to improve the bioluminescence detection by resonant excitation [193] or by electrochemical methods [194].

Finally, electro-generated chemiluminescence describes the emission of light due to electrochemical processes on the surface of an electrode, where reactive intermediates are created by an electrical potential applied to a luminophore, and this excited-state luminophore decays to its ground state under photon emission in the presence of a coupled analyte [195]. Electro-generated chemiluminescence, often based on inorganic complexes as luminophores and Pt, Au or carbon electrodes, has a high sensitivity and only very low optical background signals and is thus well-suited for many biosensing applications [196]. Another large field is DNA-based electro-generated chemiluminescence biosensors, as DNA probe molecules can hybridize with the target with high selectivity [197], while organic nanoparticles are also often used due to the possibility of assembling them from a large amount of π electron-containing organic compounds [198].

### 3.6. Photoluminescence

Photoluminescence, i.e., fluorescence and phosphorescence, describes the effect that activated molecules are excited by a specific wavelength of light and are elevated from normal energy levels to higher energy levels [199]. Fluorescence and phosphorescence are differentiated by the glowing time, where typical fluorescence lifetimes are around 10^−9^ s, while phosphorescence can continue for seconds or longer after excitation [199]. Molecules with fluorescent properties are called fluorophores. They are interesting for optical biosensors since fluorescence is usually proportional to the number of biomolecules attached [200,201]. Interestingly, functionalized fluorescent nanodiamonds can also be used to measure intracellular thermometry and magnetometry with high spatial resolution [202]. Similar to electro-generated chemiluminescence, fluorescence biosensors also often work with optical fiber platforms [203,204,205].

Phosphorescent biosensors can detect not only the wavelength but also the lifetime of the light emission. This effect was reported by Dong et al., who developed an implantable glucose monitor with an enzyme-driven phosphorescence lifetime-based glucose-sensing assay [206]. Phosphorescence lifetime was also imaged by Goncharov et al. to measure glucose concentration by an insertable sensor [207]. An important advantage of phosphorescent biosensors is the possibility of avoiding interference with spontaneous fluorescence in complex biological samples [208,209,210].

### 3.7. Interferometry

**Figure 9:**
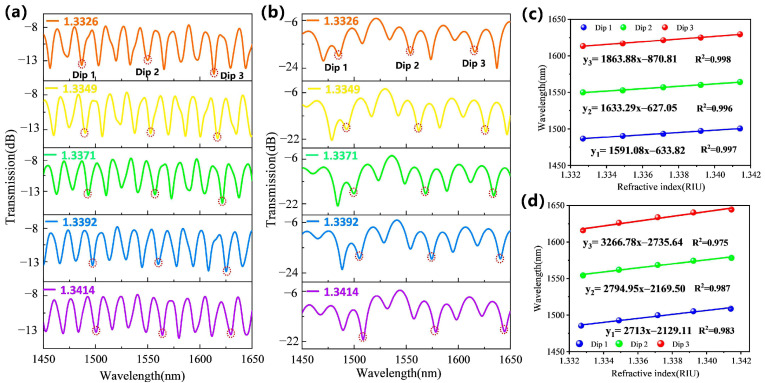
(**a**) Experimental detection spectra of the traditional tapered fiber-optic sensor (fiber waist diameter 7.25 µm) and (**b**) tapered-in-tapered fiber-optic sensor (micro-waist diameter 7.18 µm) under different refractive index (RI) environments. (**c**) RI sensitivity of the traditional tapered fiber-optic sensor and (**d**) tapered-in-tapered fiber-optic sensor. From [214], originally published under a CC-BY license.

Different interferometers, such as the Mach–Zehnder, Michelson, Fabry–Perot and Sagnac interferometers, can be used as optical biosensors, often combined with special optical fibers, e.g., polarization-maintaining, microstructured, thin-core and other fibers [211]. All interferometers work via interference between light propagating along different optical paths, resulting in a path difference that is related to the refractive index of the environment [212]. Thus, a main problem of interferometry is the selectivity toward the target analytes, which makes the functionalization of the interferometer sensing platform necessary, e.g., with antibodies, aptamers, enzymes or nucleic acids [213]. For a functionalized interferometer platform, the sensitivity for the refractive index can be further increased by geometrical optimization of the fiber-optics, as shown, e.g., in Figure 9 for the case of a Mach–Zehnder interferometer with traditional tapered and tapered-in-tapered fiber-optic sensors, with the latter showing a significantly higher sensitivity (Figure 9c,d) [214].

### 3.8. Critical Comparison of Optical Techniques Used in Optical Biosensors

Diverse performance metrics play an important role in the potential applications of optical biosensors. Generally, specific performance metrics, such as response time or dynamic range, are always connected with a specific application, making such numbers not comparable in general. Here, we thus give a qualitative comparison of the different optical techniques used in optical biosensors.

Generally, SLR is highly sensitive due to the collective coupling in periodic nanostructures, leading to sharper resonances and stronger field enhancement as compared to LSPR [215]. SERS offers a high sensitivity of the measurements as it amplifies the signals [216]. LSPR shows a high refractive index sensitivity, but a lower field confinement, as compared to SLR and SERS [217]. The other techniques, such as UV/Vis, colorimetry and polarimetry, have further reduced sensitivity.

Similarly, the limit of detection is best for SERS and SLR, while luminescence techniques depend on the efficiency of labeling. LSPR also offers a good limit of detection, but it depends on surface functionalization noise. Colorimetric systems have the lowest limit of detection, as they usually need visible changes [217].

This order changes when the dynamic range of the different methods is compared. Here, luminescence and UV/Vis are favorable, followed by LSPR and SERS, while SLR, colorimetry and polarimetry show a narrow dynamic range due to nonlinear responses at high analyte concentrations, based on surface saturation.

Reproducibility is again high in UV/Vis, colorimetry and polarimetry measurements, followed by luminescence techniques. The variability of surface chemistry in LSPR biosensors is a problem regarding reproducibility, while SERS—where the signal amplification depends on the homogeneity and reproducibility of the SERS nanotags—and SLR—depending on precise nanofabrication of periodic patterns—have the lowest reproducibility [217]. A similar order is found if costs and scalability are taken into account, where colorimetry and UV/Vis are advantageous, while SERS and SLR are again most problematic due to the necessary nanofabrication. LSPR biosensors can be produced at intermediate costs, as mass production by colloidal nanoparticles or fiber coatings is possible [218]. It should be mentioned that some techniques based on nanoparticles, such as SERS, show batch-to-batch variability due to size and shape dispersion based on small modifications of the production parameters, besides an environmental impact on the detected signals by variations in the pH value of a solution, temperature, contaminants in real samples, etc. However, colorimetric sensors based on nanoparticles have a high reproducibility, as these nanoparticles can be produced with a small standard deviation.

Miniaturization is another important point to enable the portability of these optical biosensors. In many cases, SERS and SLR require lasers and spectrometers and are thus least portable, while there are indeed portable devices available [219,220]. UV/Vis spectrometers can partly be miniaturized, enabling a certain portability. Colorimetric and LSPR-based optical biosensors can most easily be miniaturized, using fiber-optics [221]. In addition, sample preparation has to be taken into account, where SERS and SLR again are most complicated as they usually need surface functionalization and preparation of nanostructured substrates, while colorimetry and LSPR of biofluids require the lowest amount of preparation.

As this comparison shows, there is no technique that is advantageous in all cases. The high sensitivity of SERS and SLR is connected with low reproducibility, and conversely, the high reproducibility of UV/Vis and colorimetry is connected with low sensitivity [217]. Similarly, portable instruments used for colorimetry or LSPR have a relatively low sensitivity, while larger instruments like SERS and SLR offer better analytical performance. In addition, label-free techniques such as LSPR, SLR or polarimetry combine simpler sample preparation with lower specificity, while labeled detection, as often used in luminescence and SERS, needs more complex chemical sample preparation to reach the typical higher sensitivity.

## 4. Applications

Optical biosensors are used in different applications, e.g., medicine and drug discovery; forensics and detection of biological warfare agents; food safety; fermentation; and diagnosis of plant diseases. Many of these applications nowadays use smartphones for the signal presentation; thus, an overview of such applications is given here.

### 4.1. Smartphone-Based Applications

In recent years, smartphone-based applications have gained more and more interest. In many applications, smartphones are coupled to optical sensors by optical fibers, mostly polymer optical fibers made from poly (methyl methacrylate) (PMMA) that are often part of the sensor [222]. Zong et al. described different possibilities of smartphone-based optical biosensors for genetic testing, using colorimetry, fluorescence detection or microscopic imaging in order to detect different influenza viruses, hepatitis B, genetic diseases such as congenital deafness or cystic fibrosis, or cancers [223]. Beduk et al. gave an overview of smartphone-based multiplexed optical biosensing, i.e., the detection of several biomarkers in a single test, especially for medical purposes [224].

A photochemical biosensor for the determination of whole blood creatinine was developed by Cheng et al. [225]. They used a two-channel optical signal differential processing technique for the colorimetric signal of the photochemical test strip to improve the sensitivity of the system and to differentiate between whole blood creatinine and endogenous creatinine.

For the detection of uric acid with a high sensitivity and a low detection limit of 0.02 mM, Jain et al. used fiber-optic surface plasmon resonance with SiO_2_ as a dielectric layer with low refractive index [226]. The color sensitivity of the digital smartphone camera is used to detect blue and red color channels instead of the usually applied narrow-band filters for the analysis of the spectral data. The sensor was found to be stable for nearly half a year, making it suitable for point-of-care use in rural or remote areas.

Trace pollutants could be detected by a fluorescence biosensor consisting of an all-fiber optical system and a microfluidic system connected to a smartphone [204]. Using an asymmetric Y-shaped fiber coupler, transmission of excitation light and fluorescence collection could be combined. With this assay, bisphenol A and norfloxacin could be detected in 15 min with high sensitivity, while the corresponding smartphone application was able to automatically interpret the results and give a pollution warning.

**Figure 10:**
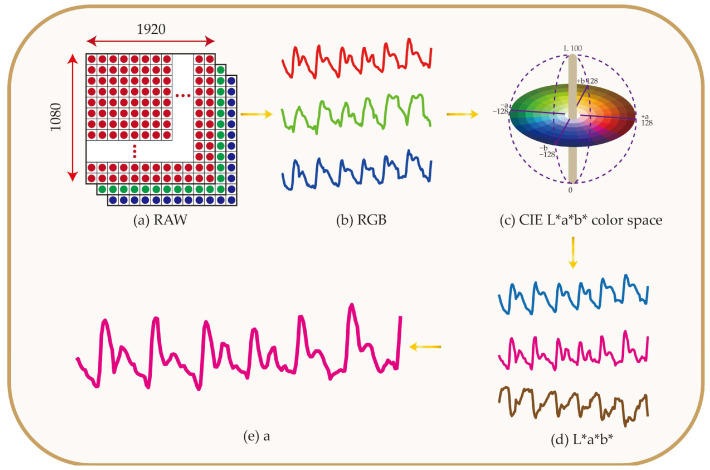
Scheme of smartphone collecting RGB channel information of the original image, converting it into L*a*b* channels, and extracting the information of channel a* for PPG calculations. (**a**) Schematic of a frame of an image. (**b**) PPG signals of red, green, and blue channels. (**c**) CIE L*a*b* color space. (**d**) PPG signals of L*a*b* channels. (**e**) PPG signal of channel a*. From [227], originally published under a CC-BY license.

Fan et al. developed an optical sensor to measure hemoglobin concentration non-invasively, using a smartphone camera [227]. The multi-wavelength LED module, combined with a specific phone fixation, enabled measuring hemoglobin concentration via L*a*b* color space transformation and using a* channel for the quantification, as shown in Figure 10 [227]. In this way, they reached *R*^2^ = 0.88, which was sufficiently accurate for non-invasive hemoglobin concentration measurements.

For SARS-CoV-2 testing, Kawasaki et al. developed crystal-film-based label-free optical sensors coupled with a light source and a smartphone or a commercial spectrometer via optical fibers [228]. They used a cycloolefin polymer-based imprinted photonic crystal film with a nanostructured hole pattern as the sensor, which was hydrophilized, silanized and incubated with glutaraldehyde before SARS-CoV-2 antibodies were immobilized on the surface. The smartphone-based spectrometric system showed a sensitivity comparable to that of the commercial spectrometer.

### 4.2. Medical Applications

Among optical biosensors, a large number are used for medical applications [229,230,231]. Thus, several authors reported improved sensors during the last few years, leading to several reviews about general or specific medical applications.

Several authors report recent advancements in optical biosensors for cancer detection, as such optical biosensors can detect cancer in a smaller number of cells and thus earlier than common techniques [230,232,233,234,235,236,237].

Wu et al. gave an overview of flexible optical biosensors for in vitro as well as in vivo measurements, measuring, e.g., physical properties like body temperature, heart rate and blood oxygen saturation as well as detecting target analytes in sweat, tears, and saliva [238]. Fiber-optic-based biosensors for precision medicine were reviewed by Luo et al., who described the advantages of miniaturized sensing area, flexible operation of light and the possibility of integrating them into wearables or even implanting them [239]. Vavrinsky et al. concentrated on optical biosensors in medical wearables and highlighted their immunity to electromagnetic interference, electrical safety and simple miniaturization [240]. A special kind of optical sensor, the fiber Bragg grating sensor, was described by Prathap and Saara, showing its broad range of applications in minimally invasive surgery, musculoskeletal sensing, as a heart rate sensor, blood pressure sensor, temperature sensor and other biosensors [241]. Optical biosensors based on surface plasmon resonance, on the other hand, were discussed by Janith et al., who mentioned the high sensitivity and specificity of this technique for the detection of biomolecular interactions [242].

Among the most recent case studies, Tene et al. reported an SPR-based optical biosensor for clinical malaria biomarker detection using a multilayer plasmonic nanostructure with angular interrogation [243]. This sensor is based on the change in the refractive index in malaria-infected blood plasma. As the authors have shown, their SPR sensor with embedded graphene, silicon nitride, and thiol-tethered ssDNA could be used for real-time, label-free, stage-specific malaria diagnosis with high sensitivity, which supports future development toward point-of-care applications.

Another SPR-based optical biosensor was suggested by Runthala et al., who prepared a multilayer sensor from a bimetallic layer (Al/Au), a dielectric layer (MgF_2_), and an optimized number of layers from 2D nanomaterial (MoS_2_) [244]. Detection accuracy, sensitivity and figure of merit were optimized by simulation with finite element methods and experimental validation. The proposed sensor revealed a high sensitivity and detection accuracy for SARS-CoV-2 and other viruses based on a change in the refractive index.

Liu et al. reported the very early detection of S100B biomarkers with a nanophotonic biosensor combined with deep learning quantification to enable mild traumatic brain injury management, measuring different biospecimens like serum, urine, and saliva [245]. They found a low detection limit of 1 pg/mL, a broad dynamic range up to 100 ng/mL, and a high accuracy of about 98%, combined with a fast turnaround time of about 30 min. In addition, nearly half of the samples could not be detected by ELISA but were detected by the developed biosensor.

As this short overview shows, there are a large number of reviews available, in addition to experimental studies, regarding general and specific medical applications of optical biosensors, which are thus not reviewed here in more detail.

### 4.3. Drug Discovery and Forensics

Forensic investigations include the accurate identification of drugs, usually for legal proceedings [246]. Among the different methods applied for this, optical sensors play an increasingly large role [247].

Raman spectrometry, as an example, was suggested by Grover et al. to identify fentanyl and other drugs by a handheld instrument [248], while Altunbeck et al. used surface-enhanced Raman spectroscopy to detect Doxorubicin and Paclitaxel [249]. The same technique was used by Mistek et al. in forensics to differentiate race by a bloodstain [250], while Almehmadi et al. used surface-enhanced Raman spectroscopy to detect peptide drugs by hyperspectral analysis of an ensemble of spectra for each sample [251].

Surface plasmonic biosensors have also been reported for the precise identification of legal or illegal drugs, as several researchers reported [13,252,253]. Another highly common method to study biomolecular interactions, as necessary in drug discovery and development, is biolayer interferometry. This technique also measures small changes in the light reflected from a sensor surface on which a layer of biomolecules is immobilized [254]. It allows for measuring the biofilm thickness on the biosensor surface [255].

### 4.4. Food Safety

Optical biosensors have also been widely used for food safety applications recently. Among the target analytes to be detected, there are toxic chemicals, heavy metals, antibiotic residues, pesticides, and pathogens [256]. A detailed description of different biosensors, especially enzyme-, antibody-, nucleic acid-, whole cell-, and molecularly imprinted polymer-based biosensors, for the detection of diverse analytes in food quality monitoring was recently provided in Ref. [257].

Mycotoxins such as the potentially carcinogenic ochratoxin-A can be detected by diverse optical biosensor techniques, such as fluorescence, SPR, colorimetry, and chemiluminescence [258]. Many of these techniques use nanophotonic-optofluidic biosensors, enabling the construction of compact devices that combine opto-electronics, microfluidics and data analysis on ultracompact chips [259].

It should be mentioned that while optical biosensors are increasingly used for food safety control, enabling on-site testing [260,261], there are still problems regarding regulatory acceptance on the one hand and transfer into commercialization on the other hand [262].

Among the recent case studies, Qiu et al. applied a label-free fiber ring laser biosensor to detect *Salmonella typhimurium* in an optical fiber interferometer with high sensitivity [263]. In this way, they could detect *S. typhimurium* in less than 20 min with a detection limit of 1 cell/mL solution. The experiments showed good stability, high sensitivity, and fast response times of the developed sensor as well as limited cross-reactivity to other bacterial species, such as *Staphylococcus aureus* and *Listeria monocytonenes*. Besides measurements in solution, *S. typhimurium* could even be detected in chicken and pickled pork samples, although with a slightly smaller wavelength shift, thus increasing the detection limit slightly.

To detect insecticides in lettuce, Iwantono et al. developed an LSPR sensor based on Au nanoparticles [264]. For the experimental investigation, lettuce plants were grown for 10 days before the insecticide Malathion was applied to the leaves and then rinsed. An optical fiber was used to transmit the light from a halogen light source (260–2500 nm) to the Au nanoparticle sample and to transmit the reflected light to a spectrometer. The authors found the expected correlation between Malathion concentration and LSPR response, stability of the sensor for 10 min and repeatability of the results.

### 4.5. Fermentation

During wine fermentation, interactions between the vessel and product may lead to inorganic or organic species migrating into the wine, which can result in toxic trace elements in the wine or other unrequired changes [25]. Besides wine, beer and kombucha also belong to the fermented beverages in which optical biosensors based on yeast as sensitive elements can be used to detect specific contaminants, spoilage organisms, or hazardous compounds during the fermentation process or in the final product [265].

For glucose detection in fermentation processes, Yang et al. suggested an NIR transflection/transmission sensor [266], with transflection describing a spectroscopic measurement produced by transmitting light through a sample and reflecting it back again through the sample onto the probe [267], and showed that the combination of transmission and transflectance flow cells in series could significantly increase the performance in complex natural media containing yeast extract and peptone [266]. On the other hand, a simple optical density measurement with a UV/Vis spectrophotometer was used to estimate the yeast biomass concentration of *Saccharomyces cerevisiae* (Baker’s yeast) during industrial xylose fermentation [268].

Other case studies are reported, e.g., by Miliutina et al., who reported a plasmon-active optical fiber sensor of enzymatic activity [269]. Their optical biosensor could estimate not only the presence of enzymes but also their activity and their suppression. For this, the optical fiber probe was covalently functionalized with enzyme-sensitive organic molecules and immersed in a solution containing different amounts of β-glucosidase at varying experimental conditions, such as different temperatures and pH values. Due to the presence of the enzyme, the local environment of the optical fiber probe was changed, resulting in a shift in the spectral position of the plasmon absorption band that was measured in reflected light.

A lab-on-chip system based on a spectroscopic biosensor, using a 400–900 nm VIS/NIR spectrometer, was suggested by Zhou [270]. They integrated chemometric modeling as well as AI-based predictive analysis to reach a high prediction accuracy for fermentation metabolites with low detection limits. The interpretability and robustness of the spectral analysis could be increased by partial least squares regression and AI machine learning models for the nonlinear relation given by the data.

### 4.6. Diagnosis of Plant Diseases

Another application in which optical biosensors are often used is plant disease detection. Plant diseases are especially important to detect in time in food plants. Proper plant disease management, based on accurate and timely diagnosis, can help reduce pesticides. Typical biomarkers used for this application include inorganic and organic small molecules, metal ions and macromolecules such as proteins and nucleic acids, while sensors can use fluorescence, SPR, chemiluminescence, coated optical fibers or optical waveguides [27]. In many cases, nanoparticles from silver, gold or chitosan, carbon nanotubes or graphene oxide are used in these biosensors [271].

Optical biosensors can also be used for quite specific examinations, such as plant-microbe interactions outside a lab, via localized surface plasmon resonance, lateral flow immunoassays with optical evaluation, bioluminescence or fluorescence sensors [272]. Plasmonic biosensors based on surface-enhanced Raman scattering were used to monitor microRNA activity in living plant samples non-invasively [273]. With infrared spectroscopy, the plant parasitic nematodes *Meloidogyne enterorlobii* could be detected much earlier than symptoms became visible [274].

In a recent case study, Dong et al. developed a heterogeneous SERS substrate from Ag/Au nanoparticles/TiO_2_/oxidized carbon cloth [275]. In this way, they could detect imidacloprid, widely used for pest control, at a very low detection limit of 4 μg/mL. Interestingly, they also mention the hydrophobic structure of the sensor material, leading to self-cleaning properties and thus enabling repeated use, as opposed to many traditional SERS substrates.

An LSPR-based optical biosensor was used to detect the Tomato Leaf Curl New Delhi Virus [276]. Virk et al. developed a D-shaped fiber-optic probe for evanescent wave absorption-mediated DNA detection of the virus. For this, they functionalized the probe with amine functional groups by a conventional amine silanization process, while the LSPR sensor surface was functionalized by gold nanoparticle immobilization and by incubating cross-linker-mediated complementary DNA. The single-stranded viral DNA (ssDNA) as a target-specific sample could be detected with a limit of approximately 85 ng/µL. Finally, the system was transferred into a portable optical sensor, enabling real-time monitoring of plant viruses from the genus *Begomovirus*.

### 4.7. Microfluidic Integration of Optical Biosensors

Several optical biosensors are used in microfluidic platforms. Lab-on-chip applications can integrate optical biosensors to allow for label-free, real-time detection in miniaturized systems [277]. Optofluidic devices enable localization of light and analyte streams at the same time, which increases sensitivity and reduces the sample volume, in this way improving point-of-care diagnostics [277]. In many cases, such lab-on-chip systems use laminar flow regimes to precisely control the interaction of the analyte with the optical sensing region [278].

Among the microfluidic systems, continuous-flow microfluidics enable real-time monitoring of biochemical processes, where integrated optical biosensors can continuously investigate the analyte with improved reproducibility and signal stability [278]. Such continuous-flow platforms are often applied for environmental monitoring, clinical diagnostics and control of bioprocesses.

Droplet-based microfluidics, on the one hand, are used for high-throughput screening as well as single-cell or single-molecule detection, using, e.g., fluorescence detection for the real-time investigation of every single droplet, which enables rapid diagnostics with low reagent consumption. On the other hand, aligning the optical readout with the moving droplets is more complicated than measuring in continuous flow [278].

Nowadays, novel approaches based on optically actuated microfluidics, i.e., light-driven flow-control, and on-chip spectroscopy and interferometry are being investigated [263]. Another approach is paper-based microfluidics, where capillary-driven flow is used, making pumps unnecessary. Such paper-based devices are inexpensive and portable and can often be used with smartphone-based evaluation [279].

Going one step further, even organ-on-chip systems have been equipped with optical biosensors. Such platforms replicate physiological microenvironments and thus necessitate the continuous monitoring of oxygen, pH value, biomarkers, metabolic activity and also cellular responses to drug delivery. Long-term monitoring can be performed by embedded optical biosensors [280], especially for use in personalized medicine and drug screening [281].

Generally, recent trends include developing hybrid systems that integrate droplet microfluidics with optical detection to simultaneously detect multiple analytes based on spectral coding to integrate microfluidics into wearables [282] and to improve the automation and integration of artificial intelligence for the real-time interpretation of data received by optical biosensors.

## 5. Future Perspectives—Commercialization, Regulatory Approval and Technical Challenges

### 5.1. Commercialization and Regulatory Approval

While the aforementioned systems have been shown to work very well in laboratory environments, there are still barriers regarding commercialization and regulatory approval, especially regarding portable biosensing platforms, as well as technical challenges.

Most importantly, biosensors are often used for medical applications, where extensive clinical trials are necessary, and their reproducibility and long-term stability have to be proven before they can be approved [283]. It is well-known that this process is time-consuming and cost-intensive, building a market barrier not only for biosensors but also for many other medical products [284]. This is especially problematic if devices are planned to be used in different regions, making it necessary to deal with different regulatory rules [283].

Even for non-medical applications, the costs of upscaling are a barrier for commercialization, as novel optical biosensors need to be either clearly technically favorable in comparison with recent standard methods or cheaper, ideally both, to have a chance in the market [283].

Biosensors often suffer from the problem of standardization. The extensive research and development of the last few years that enable more and more measurements to be performed in this way with increasing precision necessitate new types of sensors, new sensor materials, new evaluation processes, etc. While pushing the limits of biosensors further, this process on the other hand causes the problem that standardization cannot be reached as long as no final states are reached regarding calibration, validation or benchmarking of proposed optical biosensor devices, often making them hard to compare and to define as a standard [285,286,287]. In addition, new devices may necessitate new workflows or IT environments, making them harder to integrate into a clinical or another suitable environment than well-established devices and processes [10,281].

Another point scarcely mentioned is the aging of samples and sensors [114], which can be ignored in the lab, but not in a real application. In addition, real applications pose larger challenges regarding interference with other than the target molecules, as the variability of potential molecules in the sample is much larger than in a controlled lab environment [288,289,290,291]. In addition, upscaling from lab-scale to a commercial product necessitates a high reproducibility of the sensor performance, which is often not given for devices that work well in the laboratory, while upscaling reveals differences between the single sensors. On the other hand, lab-based testing instruments often need specific optical alignment that can be time-consuming and too complicated for a commercial device that should be usable by everybody [292].

Many of the aforementioned applications are based on smartphones. While this approach is comfortable and based on usually available hardware, it nevertheless poses questions regarding data security and privacy, especially if data are stored in a cloud, or if software is used that does not clearly state what happens with the potentially sensitive data of the user [293]. In addition, missing standards prohibit such smartphone-based optical biosensors from being adopted across different healthcare systems [294]. Further, different hardware, e.g., different cameras and lenses, different surroundings, e.g., varying light conditions, as well as different handling by untrained users, cause problems regarding the comparability of measurements performed with identical optical biosensors and different smartphones.

### 5.2. Technological Readiness

While some of the aforementioned techniques are already implemented in commercial systems, others are still far from being used commercially. SPR platforms have been used in commercial instruments for more than 20 years, e.g., from Biacore (Uppsala, Sweden) [295]. Other SPR platforms are technologically application-ready [296]. SPR sensors, especially fiber-based ones, are already used in diverse areas, such as clinical diagnostics, environmental monitoring or food examination [217]. Today, even nanoplasmonic systems are beginning to be used in personalized medicine and point-of-care tests [297].

Another type of already commercially used systems contains biosensors working with optical fibers, enabling their integration into wearables [298]. Optical fibers not only can be used in vivo but also enable the avoidance of interference with electromagnetic fields from the environment [299]. In addition to optical fibers, wearable plasmonic biosensors embedded in textile fabrics or other flexible substrates are already being used for continuous health monitoring of patients [300]. Generally, a shift in technology from large instruments toward miniaturized fiber-based wearable platforms can be observed.

Among the technologies that have already partly emerged from pure lab environments, optofluidic biosensors that could be used for lab-on-chip analysis should be mentioned; however, these still lack robustness, reproducibility and standardization. On the other hand, other techniques, such as nanophotonic sensors or techniques based on novel nanomaterials, are still at the laboratory stage and need further improvements regarding fabrication precision, stability, reproducibility and often sensitivity [301,302].

### 5.3. Future Perspectives

As described in the previous sub-sections, there are not only technological, but also other barriers that have recently impeded the translation from lab-scale into commercial applications. Thus, it is necessary to develop international testing standards and calibration protocols, to adopt reference materials and validation studies including several laboratories, and to integrate self-calibration and error-correction mechanisms [303,304]. Going one step further, it is necessary to set up large-scale clinical validation studies and ideally to harmonize international regulatory standards to enable translation of optical biosensors into clinical applications. To enable commercialization and market translation in general, not only regarding medical biosensors, it is further necessary to enable cost-effective fabrication, to develop user-friendly interfaces and to align with clinical workflows and market requirements.

On the other hand, miniaturization and system integration are necessary to develop robust lab-on-chip optical devices, to integrate microfluidics with photonics and electronics, and to enable scalable CMOS-compatible fabrication [304,305].

To develop data evaluation of optical biosensors further, artificial intelligence (AI)-based signal processing and noise reduction as well as pattern recognition and corresponding decision support are already being investigated [306]. While the use of AI in clinical environments can be highly supportive, it nevertheless requires comprehensible statements and decisions of the AI used to enable checking all steps by a human [303]. It is thus necessary to develop regulatory frameworks for AI-enabled diagnostics.

The aforementioned smartphone-based optical biosensors have the advantages of available cameras, light sources, and hardware but the disadvantages of inconsistent calibration, missing interoperability and standardization, and limited scalability and robustness [307], suggesting the development of solutions for these problems, as smartphones could be a good base for decentralized and personal healthcare.

Technologically, problems regarding stability, lifetime and robustness have to be solved. Temperature changes, varying humidity, and biofouling can significantly reduce the performance of optical biosensors over time [308], which is why future work must focus on the thermal and chemical stability of sensor materials as well as on anti-fouling or even self-regenerating sensor interfaces.

Generally, the future of optical biosensors offers miniaturized devices with AI-driven analytics for a digital health ecosystem, if the recent problems of standardization, reproducibility and regulatory requirements are solved.

## 6. Conclusions

Optical biosensors can be subdivided into physical and chemical biosensors, where physical biosensors are mostly used to monitor vital signs of the human body, such as ECG or skin temperature, while chemical biosensors contain a chemical receptor or recognition element and can be used for a broad range of applications. The biochemical reaction between the target analyte and the bioreceptor is converted into a readout by a transducer, such as electronic, thermal, acoustic, or optical transducers.

Concentrating on the latter, this review gave an overview of the different interactions with bio-receptors, such as photoplethysmography (PPG) in the case of physical measurements, and colorimetry, spectrophotometry, polarimetry, luminescence detection, photoluminescence or interferometry in the case of chemical biosensors.

Finally, potential applications were described, which can mainly be found in the area of biomedicine, but also in drug detection and forensics, food safety control, fermentation and diagnosis of plant diseases.

The large number of original and review papers published throughout the last few years dealing with specific techniques or areas of application underlines the importance of developing all components of optical biosensors further to improve their sensitivity, selectivity and shelf life. We hope that this overview of optical biosensors can be used as a starting point for researchers interested in this field and can stimulate new developments.

## Data Availability

No new data were created in this review.
